# Selenium, copper, zinc and hypertension: an analysis of the National Health and Nutrition Examination Survey (2011–2016)

**DOI:** 10.1186/s12872-020-01355-x

**Published:** 2020-01-31

**Authors:** Mrigendra M. Bastola, Craig Locatis, Richard Maisiak, Paul Fontelo

**Affiliations:** 1 National Library of Medicine, National Institutes of Health, Bethesda, MD USA; 2 University of Alabama at Birmingham, Birmingham, AL USA

**Keywords:** Hypertension, NHANES, Reference levels, Trace elements, Selenium, Copper, Zinc

## Abstract

**Background:**

Hypertension is a major cardiovascular illness worldwide with many underlying causes. The role of trace elements selenium, copper, and zinc in hypertension is uncertain. The objective of this study was to evaluate the role of these trace elements in hypertension.

**Method:**

Data from 6683 National Health and Nutrition Examination Survey (NHANES) participants from 2011 to 2016 were analyzed using Statistical Analytical System (SAS, version 9.4) software for the role of trace elements in hypertension in age range 8 to 80 years, irrespective of the antihypertensive medication taken. Recent American Heart Association guidelines and pediatric practice guidelines for hypertension were used.

**Results:**

Findings showed a significant positive association between serum selenium levels and hypertension but not serum zinc and copper. At optimal levels for transport and distribution, serum selenium levels of 120 μg/L or higher (reference level 70–150 μg/L) were significantly associated with hypertension (OR = 1.46, 95% CI = 1.29–1.66) after adjusting for confounding factors. At serum selenium level greater than 150 μg/L, the association with hypertension strengthened (OR = 1.69, 95% CI = 1.32–2.17).

**Conclusion:**

A positive association was found between serum selenium and hypertension, irrespective of age or anti-hypertensive medications intake. These findings also suggest that the reference levels of serum levels in healthy individuals may need to be re-determined, if supported by additional studies. If validated, patients with hypertension may also need to be cautioned about selenium intake.

## Background

Hypertension is a major cardiovascular illness, affecting more than a billion individuals worldwide and causing millions of deaths each year [1]. Although researchers have studied micronutrients such as sodium, potassium, chloride, magnesium, and calcium and their effects on hypertension, there is not much evidence available on micronutrients such as selenium, copper, and zinc [2, 3]. The roles of the trace elements selenium, copper and zinc on hypertension were analyzed using data from National Health and Nutrition Examination Survey (NHANES) participants from 2011 to 2016, following recent American Heart Association (AHA) guidelines for blood pressure categories for adult hypertension and revised pediatric hypertension guidelines for pediatric hypertension [4–6].

Selenium is an essential trace element. It is a cofactor required for glutathione peroxidase, an enzyme that protects the body against reactive oxygen species and free radical-mediated cell membrane damage. The Institute of Medicine (IOM) recommended daily allowance for selenium for both men and women is 55 μg/day [7]. In a recently published NHANES study, the average daily selenium intake in U.S. population was more than 100 micrograms [8], suggesting an intake level much higher than required, with some authors implicating high selenium in the soil as a possible reason [7, 8].

Selenium deficiencies related to total parenteral nutrition has been linked to intramural fibrosis of cardiac muscles [9]. In a recently published NHANES and Canadian Health Measure Survey based study, circulating selenium has also been found to be inversely associated with prevalence of stroke [10]. Another recent study reported that a low selenium concentrations measured in toenail samples was associated with increased risk of hypertension in Chinese adults [11].

Higher selenium levels have been associated with diseases such as hypertension, hypercholesterolemia and diabetes mellitus. A recent longitudinal study suggests that selenium may have a harmful role in the development of hypertension in the elderly [12]. High selenium levels have been associated with high serum cholesterol levels [13], and increased risk for diabetes mellitus [14, 15]. A previous study on dietary selenium intake in 2638 NHANES participants revealed a positive association of increments in serum selenium and blood pressure in the U.S. population [16]. However, no recent studies with a large number of participants address the role of selenium on hypertension or in younger people, using the revised AHA guidelines for adults and revised practice guidelines for pediatric hypertension.

Zinc has a role in cell division and enhances the action of insulin, but only a few publications discuss its role in blood pressure. In some animal studies, the role of zinc in hypertension is conflicting, with some studies suggesting higher levels, while others suggesting lower levels causing hypertension. Tubek et al. suggested alternations in zinc metabolism where zinc might be absorbed and excreted more in urine with hypertension [17]. Kim et al. showed a negative correlation between zinc and systolic blood pressure, and that serum and urinary concentrations of zinc were not significantly associated with blood pressure [18]. A study in an animal model suggested that excessive zinc intake increases systemic blood pressure and decreases renal blood flow [19]. However, inverse correlations of blood pressure and serum zinc have also been observed [20]. A recently published article has implicated zinc deficiency to hypertension in animal models [21]. In another study, excessive zinc intake elevated systemic blood pressure levels in animal models and was presumably associated with the oxidative stress [22]. Dietary zinc was inversely associated with the systolic blood pressure in young obese women, but both serum and urinary zinc concentrations were not found to be correlated with either systolic or diastolic blood pressure after adjustments to dietary intake [18].

Copper is an essential component for lysyl oxidase and superoxide dismutase enzymes, which are involved in collagen and elastin and free radical metabolism needed for healthy arteries [23]. The role of copper in hypertension is inconclusive, with some studies showing links to hypertension [20, 24] while others do not [25, 26]. Copper has been found to inhibit the activity of angiotensin converting enzyme, a key enzyme for blood pressure regulation, and a study found low blood copper levels in hypertensive group compared to normal controls in animal model [27]. Patients with moderate hypertension were associated with marginal copper deficiency in a study [28], but others reported no association of copper with hypertension after adjustment for confounding factors [29].

In summary, there is little, outdated and conflicting evidence on the effect of trace elements Selenium, Copper and Zinc in hypertension till date. The study aims to evaluate the role of these trace elements in hypertension, which can contribute towards future studies on public heath, nutrition and clinical practices regarding the safety of these trace elements.

## Methods

### Study design and population characteristics

The NHANES 2011–2016 database on trace elements having 31,522 total participants was the study’s data source. After excluding entries with missing data sets, 6683 participants were included. The study population consisted of 3289 males, 3394 females. Of these, 976 were smokers, 49 were pregnant and 1314 were on blood pressure lowering medications. NHANES categories were used to classify participants by reported race. There were 1060 Hispanics, 2338 Caucasians, 1465 Blacks, 789 Asians and 744 were in “another Hispanic” category.

### Data collection and processing

Blood pressure was calculated as the average of the three subsequent observations for systolic and diastolic blood pressure, irrespective of the anti-hypertensive medication status of the participants. Serum levels of the trace elements in the NHANES participants were measured by inductively coupled plasma-dynamic reaction cell-mass spectrometry (ICP-DRC-MS), using gallium as the internal standard.

The lower limit of detection (LLOD, in μg/dL) implemented for serum selenium, copper and zinc were set at 4.5, 2.5 and 2.9 respectively. The data were reviewed, and incomplete data or improbable values were sent to the performing laboratory for confirmation by NHANES. Only data from consented participants was used in this study. The published NHANES datasets used in this study included serum levels of trace elements, serum cholesterol, demographics, reported dietary habits and physical examination.

### Outcome assessment

The normal reference range of selenium, zinc and copper from US based medical laboratories were reviewed, and the normal clinical values for trace elements published by Mayo Clinical Laboratories (2019) were selected as reference values for this study. According to its reference website for recent lab values, the normal serum selenium, zinc and copper values were 75 to 150 μg/L; 75 to 145 μg/dL; 65 to 105 μg/dL respectively, for the age range of 8–80 years [30–32]. The reference values were used as cutoffs in analysis for all trace elements. Additionally, high normal selenium was defined as serum selenium values more or equal to 120 μg/L, where serum selenium is reported to have its optimal physiological activity [33].

In accordance with the American Heart Association 2018 guidelines, hypertension was defined as either having a diastolic > 79 mmHg or systolic > 129 mmHg for ages 13 or above [5]. For the age range 8 to 12 years, having a systolic or diastolic above the 95th percentile in accordance with the age, gender, and height was classified as hypertensive in accordance to the recently published guidelines on pediatric hypertension [6].

### Statistical analysis

Statistical Analysis System (SAS, version 9.3) software was used for data analysis. The trace elements were characterized in mean values, maximum, minimum and median values. Odds ratios (OR) with Wald confidence limits, quantile regression and polynomial logistic regression with adjustments for confounders were sequentially used in the data analysis.

All quantiles of serum trace elements were screened for possible association with hypertension, dose response curves of trace elements with occurrence of hypertension were plotted (Fig. 2). Un-adjusted Odds ratios for the demographic variables were represented in forest plots as obtained from logistic regressions. Also, the highest and lowest quantiles of the serum trace elements were analyzed with logistic regression and Odds ratios were calculated. A quantile regression model was selected to observe the effects of serum selenium on each quantile of the heterogeneous study population, since only the higher quantiles in serum selenium showed association with hypertension among the trace elements under study.

## Results

### Study participants characteristics

Median values of the study participants were age 38 years, body mass index (BMI) 26.4, and waist circumference 91.7 cm. The median serum copper level was 113.6 μg/L, median selenium level 126.1 μg/dL and the median serum zinc level was 80.7 μg/dL (Table 1). Other demographics, blood pressure and laboratory parameters relevant to this study are shown in Table 1.

**Table 1:** Summary statistics of relevant variables in the study population (NHANES, 2011–2016 data)

Variable	Mean	Standard deviation	Minimum	Maximum	Median
Age (Years)	39.3	21.96	8	80	38
BMI (kg/m2)	27	7.86	12.4	77.5	26.4
Waist circumference (cm)	89.9	25.9	25.89	177.9	91.7
SBP (mmHg)	119.3	18.05	64.67	231.3	116
DBP (mmHg)	66.2	14.42	9	116.7	67.3
Total cholesterol intake (mg/day)	282	225	1	2007	214
Total sodium intake (mg/day)	3392	1771	7	17,070	3063
Total serum cholesterol (mg/dL)	181.8	41.1	75	463	178
Serum Copper (μg/dL)	118.2	29.82	24.7	306.6	113.6
Serum Selenium (μg/L)	127.5	17.76	58.1	299.1	126.1
Serum Zinc (μg/dL)	81.76	15.16	31.4	232.5	80.7

### Unadjusted odds ratios of relevant variables and the trace elements with hypertension

Lowest and highest quantiles for each of the trace elements were screened for their association with hypertension. The lowest quantile of serum selenium and serum copper negatively correlated with hypertension, while the highest quantile of serum selenium was positively correlated with hypertension (Table 2).

**Table 2:** Unadjusted Odds ratios of the lowest quantiles and the highest quantiles of serum trace elements with hypertension. **Significant *p* < 0.01 * Significant *p* < 0.05

	Odds Ratios with 95% Confidence Interval	
	Lowest quantile	Highest quantile
Serum Selenium	0.7 ** (0.612–0.82)	1.19 ** (1.02–1.39)
Serum Copper	0.99 (0.85–1.16)	1.25* (1.07–1.46)
Serum Zinc	1.07 (0.93–1.26)	1.11 (0.96–1.29)

**Fig. 1:**
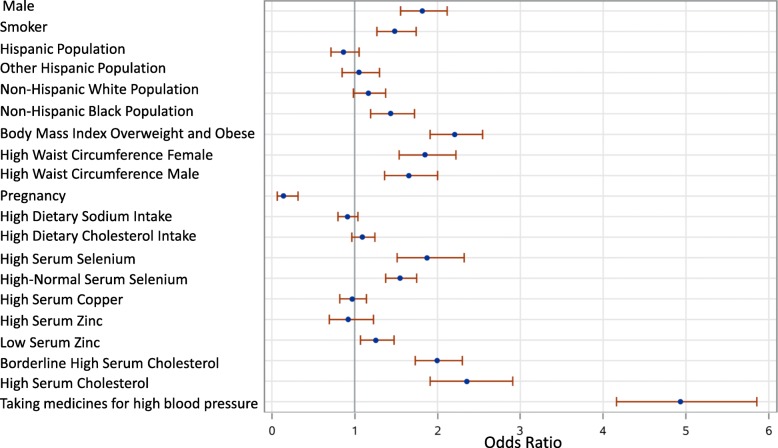
Unadjusted Odds ratios (95% CI) for hypertension with serum trace elements, demographic, dietary and lab variables

**Fig. 2:**
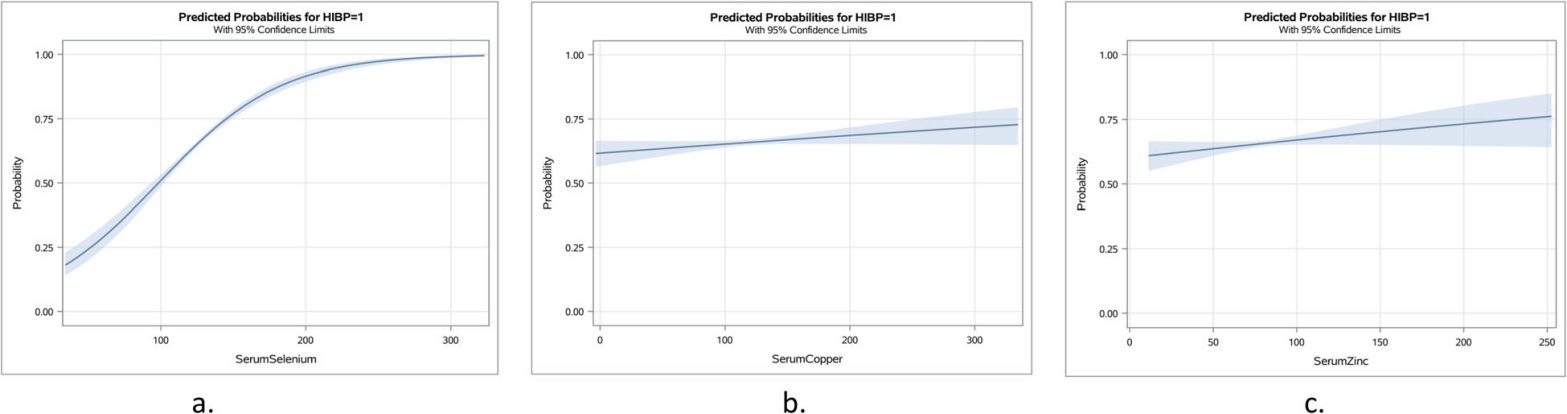
Estimated probabilities of hypertension at increasing levels of serum selenium (**a**), serum copper (**b**) and serum zinc (**c**)

Unadjusted Odds ratios were calculated for each of the relevant variables for hypertension causation and the serum trace elements (Table 2). The results of the unadjusted Odds ratios with 95% confidence intervals (CI) are shown in Fig. 1. Estimated probabilities comparisons of serum trace elements selenium, zinc and copper are shown in Fig. 2. Increased probability for hypertension was noted only in case of high serum selenium levels.

### Predicted probabilities for the serum trace elements with hypertension

The predicted probabilities of serum selenium, serum copper and serum zinc as an output from logistic regression analysis revealed no significant association of serum copper and serum zinc levels with hypertension (Fig. 2). However, there was a significant positive association of high serum selenium with high blood pressure (Table 2, Figs. 1 and 2). These initial findings of association concurred with subsequent analyses.

### Confounding factors adjustments with polynomial regression models

Confounding factors considered for multinomial logistic regression model were age, smoker, gender (male), BMI > 24, borderline high cholesterol, high serum cholesterol, high waist circumference for both males and females (defined as > 102 cm for males and > 88 cm for females), high daily sodium intake (defined as > 2300 mg/day), and daily cholesterol intake and taking anti-hypertensive medications (Fig. 1). Among the different races, being in the Black population of non-Hispanic origin was considered as a confounding factor in the analysis, but pregnancy was not (Fig. 1).

After adjusting for confounders, serum selenium levels of 120 μg/L or higher (reference level 75–150 μg/L) were significantly associated with high blood pressure (OR = 1.46, 95% CI = 1.29–1.66). Also, at serum selenium greater than 150 μg/L, the association with high blood pressure strengthened (OR = 1.69, 95% CI = 1.32–2.17) even after adjustment for confounding factors (Table 3). The adjusted odds ratios for hypertension with serum selenium at highest quantile versus lowest quantiles were significant at 1.19 (95% CI = 1.02–1.39) and 0.7(95% CI = 0.612–0.82) respectively.

**Table 3:** Odds ratios for hypertension and serum selenium, serum copper and serum zinc levels adjusted for confounding factors. High and low values refer to normal lab range values for trace elements

	Analysis of Maximum Likelihood Estimates			Odds Ratio Estimates		
	Standard Error	Wald Chi-Square	Pr > ChiSq	Point Estimate	95% Confidence Limits	
High Serum Selenium (>150μg/L)	0.13	17.12	< 0.01	1.69*	1.32	2.17
High Normal Serum Selenium (>120μg/L)	0.07	33.54	< 0.01	1.46*	1.29	1.66
High Serum Copper (>145μg/dL)	0.09	0.34	0.68	0.95	0.79	1.14
High Serum Zinc (>105μg/dL)	0.13	0.23	0.33	1.06	0.83	1.37
Low Serum Copper (<75μg/dL)	0.2	0.02	0.89	1.027	0.7	1.5
Low Serum Zinc(<65μg/dL)	0.09	0.11	0.89	1.21	1.04	1.43
Serum Selenium(Q1)	0.04	44.2	< 0.01	0.63*	0.55	0.72
Serum Selenium(Q4)	0.03	18.42	< 0.01	1.34*	1.17	1.53

*Q1* lowest quantile, *Q4* highest quantile. *Significant at *p* < 0.01

The adjusted odds ratios for hypertension with serum zinc at highest versus lowest quantiles were not significant at 1.11(95% CI = 0.96–1.29) and 1.07(95% CI = 0.93–1.26) respectively. For serum copper, the adjusted ratios of hypertension at highest versus lowest quantiles were at 0.99(95%CI = 0.85–1.16) and 1.25(95%CI = 1.07–1.46) respectively. However, subsequent logistic regression analysis for higher or lower lab reference values of serum copper did not yield significant results for hypertension. The adjusted odds ratios for lower and higher than reference serum copper value were both not significant at 1.03 (95% CI = 0.7–1.5) and 0.95(95% CI = 0.79–1.14) respectively.

**Fig. 3:**
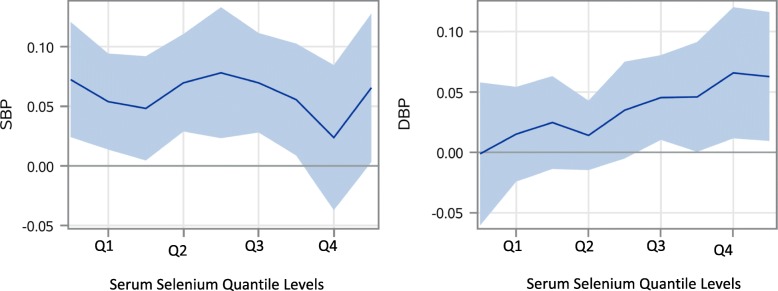
Estimated parameter plots with 95% CI for systolic (SBP) and diastolic (DBP) blood pressures for the four quantiles of serum selenium. (Q1 = < 115.9 μg/L, Q2 = 116–126.1 μg/L, Q3 = 126.2–137.2 μg/L, Q4 = 137.3- < 299.1 μg/L)

**Fig. 4:**
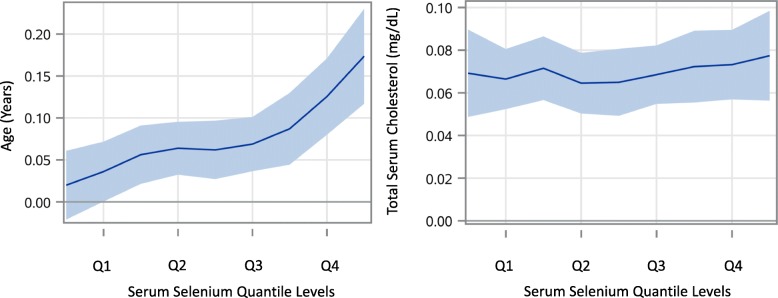
Estimated parameter levels with 95% CI by quantile levels (Q1–4) of serum selenium and its effects on incremental age and total serum cholesterol (Q1 = < 115.9 μg/L, Q2 = 116–126.1 μg/L, Q3 = 126.2–137.2 μg/L, Q4 = 137.3- < 299.1 μg/L)

Since the association of increments in serum selenium was not linear or uniform at all serum selenium levels and the association with hypertension increased from values at 150μg/L compared to 120 μg/L (Fig. 2 and Table 3), quantile regression models were also analyzed for serum selenium, systolic blood pressure, diastolic blood pressure, age of the participants and total serum cholesterol levels (Figs. 3 and 4). Looking closer at the effect of serum selenium on systolic and diastolic blood pressure, the results of quantile regression revealed stronger increments in associations of diastolic blood pressure with the higher quantiles of selenium compared to systolic blood pressure, where the strength of association remained mostly uniform in all the quantiles of serum selenium (Fig. 3). Also, the quantile regression models for serum levels of selenium showed a positive association on all the quantiles of serum selenium with increments of participant age, and a stable but stronger association with total serum cholesterol levels persisted at all quantiles of serum selenium (Fig. 4), as some studies suggest [13].

## Discussion

Findings show that the higher values of serum selenium, including those in the high normal range, are associated with hypertension, but both the high and low levels of trace elements copper or zinc are not. The association of high serum selenium levels with hypertension persisted after adjustment of various confounding factors. In addition, the results of quantile regression indicate that the effect of increments in serum selenium on hypertension was stronger with diastolic blood pressure compared to systolic blood pressure (Figs. 2 and 3).

This study’s findings support previous studies reporting higher daily selenium intake in U.S. population than the rest of the world. Its results extend those of an earlier NHANES study (2003–2004), showing a positive association of serum selenium with hypertension [16], by having a larger sample size, a later and longer time period, a more inclusive age range of 8 to 80 years, accounting for potential confounding factors, and using the precise evaluation of cut off points in serum trace elements in more recent and clinically relevant AHA hypertension guidelines. The association of higher serum selenium levels, irrespective of the participants' anti-hypertensive medicine intake further strengthens the association.

Findings show higher levels of serum selenium are associated with hypertension, including the high normal range, but not associated with either high or low levels of copper and zinc. Also, the results of quantile regression indicate that the effect of per unit increments in serum selenium on hypertension was stronger with diastolic blood pressure compared to systolic blood pressure (Figs. 2 and 3). This finding is comparable with the findings by Mark et al. where a group in a nutritionally deprived population was supplemented with dietary selenium and the population developed diastolic but not systolic hypertension [34]. The finding that the increments in selenium values were observed with stable increments in total serum cholesterol over all the quantiles of serum selenium suggests the possibility of selenium accumulation with consumption of high cholesterol containing food, which are also good sources of selenium, such as eggs and meat, causing uniform association with hypertension, in both the serum selenium and serum cholesterol concurrently [35]. Also, the selenium association may be stronger than indicated, since it was found irrespective of using hypertensive medication.

Although several of the published studies suggested physiological role of serum copper and zinc with hypertension, this study did not show any correlation of hypertension with serum copper and serum zinc [18–22, 24, 27–29]. The incidental finding of the association of the low serum copper with hypertension on lower quantile versus higher quantile was not proven by subsequent polynomial logistic regression procedure after adjusting for confounding factors. However, a larger sample size could reveal different results with more focused and controlled studies. More cellular and animal models are needed in serum copper and serum zinc studies to ascertain their precise physiology and their role in blood pressure regulation. Moreover, it is essential to study the physiological effects of these trace elements on hypertension because several commercially available nutritional supplements include these trace elements in various proportions, which might lead to inadvertent effects in blood pressure in hypertensive population taking such nutritional supplements with high amounts of trace elements.

Although not the main objective of the study, the analysis also found a significant positive correlation of high blood pressure among smokers and males while there was a significant negative correlation of hypertension with pregnancy (Fig. 1). Also, high sodium intake was not associated with hypertension as expected, presumably because normotensives were consuming high sodium diets compared to hypertensives, who were probably restricting their sodium intake [36]. Studies showed that the total parenteral nutrition patients in hospitals and chronic malnutrition are more likely to develop selenium deficiency which could be replenished by food or supplements rich in selenium, such as mushrooms, garlic, asparagus, eggs and sea-salt [35, 37]. Since there are few publications about the metabolism and excretion of selenium, no definitive predictions can be made regarding its physiology and excretion mechanism. Therefore, avoiding selenium sources in food and water is the only method advisable to gain lower levels of selenium in blood. More focused and controlled studies including animal models need to be done to confirm the pathogenesis of hypertension linked with higher blood selenium levels at a molecular and cellular level.

## Limitations

One limitation of this study was defining hypertensive cases solely according to their blood pressure at examination, irrespective of their hypertension medications intake which could mask high blood pressure. We chose not to use such intake, as either an inclusion or exclusion criterion, since there are other medical conditions where anti-hypertensive medications are taken, such as beta blockers in hyperthyroidism, calcium channel blockers in arrythmias, diuretics and angiotensin-converting enzyme (ACE) inhibitors in heart failure and renal conditions and others. Also, the data on the antihypertensives medicines intake is highly subjective, with a wide range of possibilities of error, such as, intake of herbal or over counter medications, issues with compliance, issues with dosage or skipped medications on the day of examination. A recent randomized trial study suggested that these group of hypertensive medications can alter the serum levels of trace elements in a randomized trial, which makes it a confounder in our study as well, since these drugs are potentially altering serum trace elements levels [38]. For these reasons, authors opted for one objective criterion – the recent AHA guidelines for clinical hypertension diagnosis. However, any participant with usually normal blood pressure would have been included as hypertensive, if blood pressure values were temporarily high during the examinations. The effect of hypertension medication intake was statistically adjusted in multivariate analysis procedures. Although a recent publication showed the protective role of selenium for stroke, the role of selenium as a protective factor for stroke despite its association with hypertension is still unanswered [10].

## Conclusion

The study suggests that higher values of serum selenium including the high normal values may be associated with hypertension. These findings require confirmation from larger population studies so that the hypertensives may be advised to lower their daily selenium intake. The current reference levels of serum selenium may need to be redetermined if these study results are further validated.

## Data Availability

The data used in this study is publicly available and downloadable from Centers for Disease Control and Prevention website, under National Health and Nutrition Examination Survey 2011–2016 sections. Additionally, the datasets used and data analysis output from SAS are available from the corresponding author upon reasonable request. Datasets used in this study are available at: https://wwwn.cdc.gov/Nchs/Nhanes/ for demographics and dietary data under 2011 to 2016 sections. Serum trace elements lab data are directly available from https://wwwn.cdc.gov/Nchs/Nhanes/2011-2012/CUSEZN_G.XPT, https://wwwn.cdc.gov/Nchs/Nhanes/2013-2014/CUSEZN_H.XPT and https://wwwn.cdc.gov/Nchs/Nhanes/2015-2016/CUSEZN_I.XPT.

## Abbreviations

ACE: Angiotensin-converting enzyme
AHA: American Heart Association
BMI: Body mass index
CI: Confidence interval
DBP: Diastolic blood pressure
NHANES: National Health and nutrition examination survey
OR: Odds ratio
Q1–4: Quantiles 1 to 4
SBP: Systolic blood pressure
